# The Role of O-Antigen in LPS-Induced Activation of Human NK Cells

**DOI:** 10.1155/2019/3062754

**Published:** 2019-05-20

**Authors:** Leonid M. Kanevskiy, Sofya A. Erokhina, Maria A. Streltsova, Rustam H. Ziganshin, William G. Telford, Alexander M. Sapozhnikov, Elena I. Kovalenko

**Affiliations:** ^1^Laboratory of Cell Interactions, Department of Immunology, Shemyakin-Ovchinnikov Institute of Bioorganic Chemistry, Russian Academy of Sciences, ul. Miklukho-Maklaya 16/10, Moscow, 117997, Russia; ^2^Laboratory of Proteomics, Department of Peptide and Protein Technologies, Shemyakin-Ovchinnikov Institute of Bioorganic Chemistry, Russian Academy of Sciences, ul. Miklukho-Maklaya 16/10, Moscow, 117997, Russia; ^3^Experimental Transplantation and Immunology Branch, National Cancer Institute, National Institutes of Health, 20892-1203 Bethesda, MD, USA

## Abstract

NK cells can be stimulated by bacterial lipopolysaccharides (LPS). Unlike macrophages, human NK cells do not express or express very low level of surface TLR4 receptor normally required for the LPS stimulation. This has led to the assumption that the mechanisms of stimulating action of LPS on macrophages and NK cells differs. In this work, we investigated the effects of different forms of *E. coli* LPS, including mutants lacking O-antigen structures, and deacylated LPS on IFN*γ* production by purified human NK cells. The main findings were the following: (1) NK cells were more sensitive to the S-forms of LPS than the R-forms (LPS lacking O-antigen); (2) LPS triggered a significant increase in IFN*γ* production by NK cells in concentrations about 1000 times higher than those that can induce cytokine production by macrophages; (3) the composition and structure of saccharide part of LPS have a strong influence on its observed effects on NK cells; and (4) LPS fully retained the ability to trigger cytokine production in NK cells in serum-free media. The acquired data demonstrated that the presence and structure of O-antigen affects the LPS-induced activation of human NK cells.

## 1. Introduction

NK cells take part in systemic inflammatory reactions, induced by bacterial lipopolysaccharides, i.e., sepsis (reviewed in [1, 2]). In addition to the activation caused by stimulating interactions with TLR4-bearing cells (dendritic cells and macrophages), NK cells have been shown to be independently activated by bacterial LPS in the presence of cytokines, such as IL-2 [3]. The mechanism of the LPS-induced triggering of NK cell activity remains unclear. However, there is evidence that it can be independent of the TLR4 receptor complex on the cell surface [3–5], suggesting that conditions of LPS-induced stimulation of NK cells can differ from the classical model.

**Figure 1:**
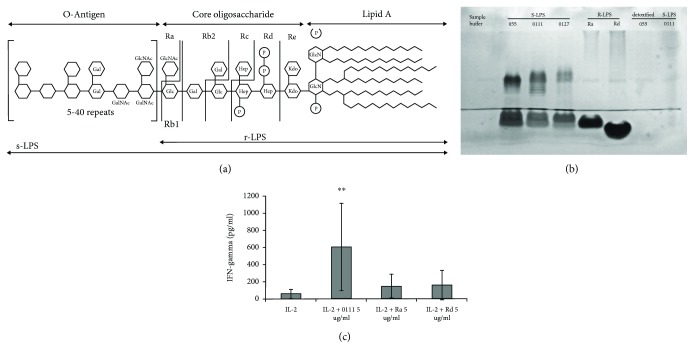
The LPS structure and the effects of the R- and S-forms on IFN*γ* production by NK cells. (a) The scheme of the LPS structure. The frontier between inner and outer core is marked by a bold line. The scheme has been taken from Bagheri et al. [14]. (b) Analysis of LPS samples by SDS-PAGE followed by silver staining. All samples were applied on gel in a concentration of 1 *μ*g/lane. (c) The effects of S- and R-LPS on IFN*γ* production by NK cells. IL-2 (500 U/ml) was added to the cell culture together with LPSs. Data for eight experiments performed on NK cells isolated from blood samples taken from different donors, in triplicate wells, are shown. Here and after data are mean ± SD and asterisks denote significant differences (^∗^ *P* < 0.05; ^∗∗^ *P* < 0.01) from control samples with IL-2 only.

The LPS structure is well characterized and described elsewhere [6], see also Figure 1(a). The LPS molecule consists of a lipid moiety, named lipid A, the core oligosaccharide and a polysaccharide tail of inconstant length (O-antigen). The lipid A is a common constituent for all strains of *E. coli*. It is made up of two glucosamine-phosphates with up to six fatty acid chains attached to both saccharide residues. The core oligosaccharide of *E. coli* LPS includes the so-called inner core (proximal to the lipid A) and the outer core fragments; it consists of 11-12 monosaccharide residues. There are 5 variants of outer core structures, named R1, R2, R3, R4, and K12, differing between *E. coli* strains [7]. The O-antigen is polymeric; it usually consists of 10–25 oligosaccharide repeating units, connected sequentially to the outer core fragment [8]. O-Antigen repeating units are specific for a current strain of bacteria and usually consist of 2-7 monosaccharide residues; most of the types of the repeating units are branched. Currently, more than 180 variants of the *E. coli* O-antigens are described [8]. There are mutant strains of *E. coli* producing truncated forms of LPS molecules, called R-forms or R-LPS, lacking the O-antigen and, in some cases, the fragments of core oligosaccharide. To indicate the structure of mutant forms of LPS, the small letters are used: Ra-, Rb-, etc. (Figure 1(a)). The normal wild-type LPS is also called S-form or S-LPS.

The lipid A fragment is responsible for most of the toxic effects of LPS. The lipid A is a ligand for the TLR4 receptor complex; this fragment alone is sufficient to induce TLR4 signaling. It interacts with the TLR4 complex via fatty acyl chains, which are strictly required for TLR4 signaling [9]. The saccharide part of LPS molecule appears to have much less immunomodulatory activity. The presence or absence of O-antigen in LPS molecule has been shown to influence the monocyte/macrophage activation via TLR4: S-LPS can effectively stimulate these cells only in the presence of LPS-binding protein (LBP) in culture media. R-LPS forms, in their turn, are able to activate macrophages in serum-free media (in the absence of LBP) [10, 11].

In this work, we have investigated the effects of different forms of LPS on IFN*γ* production by human NK cells. The obtained results revealed some differences in conditions of NK cell activation induced by LPS in comparison with the classical model.

## 2. Materials and Methods

### 2.1. Isolation of NK Cells

Blood samples were taken from healthy adult volunteers who have given the informed consent for their blood to be used in this study. The protocol was approved by the ethics committee of the Russian State Medical University. Peripheral blood mononuclear cells (PBMC) were isolated by centrifugation on Ficoll density gradients 1.077 (Paneco, Russia). Then, magnetic separation of NK cells was performed using the NK cell isolation kit (cat. #130-092-657, Miltenyi Biotec, Germany) with LD columns (cat. #130-042-901, Miltenyi Biotec). The percentage of CD3^–^CD56^+^ cells in the preparations after separation was no less than 97%. In some experiments, NK cell subpopulations were isolated from magnetically separated NK cells by fluorescent-activated cell sorting.

### 2.2. Lipopolysaccharides Used for Stimulation of NK Cells

Isolated NK cells were cultured in the medium RPMI-1640 (Paneco, Russia) supplemented with 10% FCS (HyClone, USA) at a cell concentration of 1.5 × 10^6^ cells/ml or 0.5 × 10^6^ cells/ml (for FACS-sorted cells) for 18 h in 96 U-well plates (SPL, Korea). Recombinant human IL-2 purchased from Hoffmann-La Roche (500 U/ml) was added to the cell culture. The dose of IL-2 has been optimized in our previous work [3]. The following *E. coli* LPS preparations were used: (1) full length S-LPS from strains O55:B5, O111:B4, and O127:B8 (#L4524, #L3024, and #L5024, respectively, purchased from MilliporeSigma, USA); (2) Ra- and Rd-LPS mutant forms (#L9641 and #L6893, respectively, MilliporeSigma); and (3) S-LPS with partially removed alkaline hydrolysis fatty acid portions (detoxified S-LPS) from strains O55:B5 and O111:B4 (#L9023 and #L3023, respectively, MilliporeSigma). Results of analysis of LPS preparations provided by the manufacturer are presented in Supplementary Data (available here). LPS preparations were added to isolated NK cells in concentration 5 *μ*g/ml. After incubation, cell-free supernatants were collected for estimation of cytokine production. For serum-free experiments, NK cells were incubated in AIM-V serum-free medium (cat. #12055-083, Gibco, Thermo Scientific, USA).

### 2.3. IFN*γ* Production Measurement

IFN*γ* amount in supernatants was analyzed using the IFN*γ* ELISA kit (Vector-Best, Russia). Plates were read using a Multiskan FC plate reader (Thermo Fisher Scientific, USA) set to 450 nm absorption wavelength with reference wavelength of 620 nm.

### 2.4. Electrophoretic Analysis of LPS Preparations

The quality of LPS preparations was checked by electrophoresis (SDS-PAGE) following silver staining of gels (see Figure 1(b)). The procedure was performed according to the protocol presented by Tsai and Frasch [12]. The exact mechanism of silver staining of LPS is not fully understood. However, it is believed that silver ions interact with fatty acid chains of lipid A fragment rather than with saccharide residues [13].

### 2.5. Flow Cytometry Analysis

Anti-CD14-PerCP (BD Pharmingen, USA), anti-CD3-PE (Dako, USA), and anti-CD56-APC (Miltenyi Biotec) were used for the analysis of purity of isolated NK cells. Samples were analyzed by flow cytometry (FACSCalibur, BD Biosciences, USA). The flow cytometry data were analyzed using FlowJo software version 7.6 (TreeStar, Ashland, OR, USA) and Flowing Software version 2.0 (Dr. Perttu Terho, Finland).

### 2.6. Cell Sorting

The cell sorting of NK cell subpopulations was performed on the FACSVantage DiVa (BD Biosciences, San Jose, CA, USA). The following antibodies were used: anti-CD3-PC7, anti-CD56-APC (both from Beckman Coulter, USA), anti-CD57-FITC (eBioscience, USA), and anti-HLA-DR-PE (Sony Biotechnology, Japan).

### 2.7. Mass Spectrometry

MALDI-TOF MS analyses of the LPS samples were performed using Ultraflex TOF/TOF mass spectrometer (Bruker Daltonics, Germany). Mass spectra were recorded in linear mode of negatively charged ions in the *m*/*z* range of 500-5000 daltons using 2,5-dihydroxybenzoic acid (20 mg/ml, acetonitrile/0.1% TFA 1 : 1, *v*/*v*) as a matrix. The MS data were processed using BrukerDaltonics Flex Analysis 2.4 software. Results of analysis are presented in Supplementary Data.

### 2.8. Statistics

Data were analyzed with the SigmaPlot program, version 11.0 (Systat Software Inc., USA). For estimation of differences between two groups of data, Student's *t*-test was used. Values of *P* < 0.05 were considered statistically significant.

## 3. Results

### 3.1. S-LPS More Effectively Activates NK Cells than R-LPS

First, we have compared the effects of R- and S-LPS on IFN*γ* production by magnetically separated NK cells. NK cells were incubated in the presence of IL-2 and S-LPS (strain O111) or Ra- or Rd-LPS (see Figures 1(a) and 1(b)). The optimal doses of IL-2 (500 U/ml) and LPS (5 *μ*g/ml) for LPS-induced NK cell stimulation had been determined in our previous work [3]. The S-LPS demonstrated the marked positive effect on IFN*γ* production by NK cells (Figure 1(c)). In their turn, the R-forms of LPS did not significantly increase IFN*γ* production. It is important to note here that Ra- and Rd-LPS preparations have homogenous structure, but S-LPS preparations contain a mix of molecules differing by the amount of oligosaccharide repeating units in their O-antigen. Some molecules in S-LPS preparations are in fact Ra-LPS. Some molecules contain only one repeating unit; they are sometimes denoted in literature as SR-LPS (semi-rough). A large portion of the molecules has a long polysaccharide chain containing different but significant numbers of repeating units; they form a characteristic pattern in PAGE gel (Figure 1(b)). So, we can conclude that the observed LPS-induced stimulation of the IFN*γ* production in NK cells seemed to be mainly due to the mix of O-antigen containing molecules in the S-LPS preparations. Thus, the presence of O-antigen, probably, plays an important role in the NK cell stimulation by LPS.

### 3.2. S-Forms of LPS Stimulate NK Cell IFN*γ* Production in Serum-Free Media

**Figure 2:**
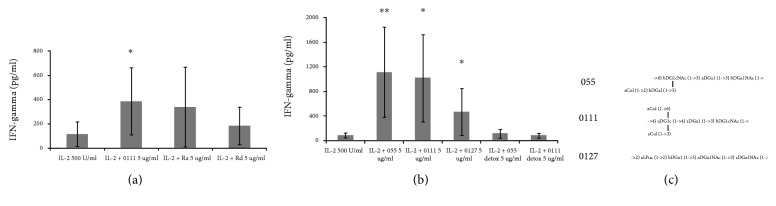
The effects of the different LPS forms on IFN*γ* production in serum-containing and serum-free media. (a) The effects of S- and R-LPS on the IFN*γ* production in AIM-V serum-free media. Results from six independent experiments are shown. (b) The effects of normal and deacylated S-LPS from different *E.coli* strains on IFN*γ* production by NK cells. (c) The structures of repeating units of O-antigen of LPSs used in this work. Results from four independent experiments are shown.

So, we have found that preparations of the S-forms of LPS increased IFN*γ* production in NK cells. These experiments have been performed using the RPMI-1640 culture media supplemented with fetal calf serum (FCS). A critical feature of the LPS-induced activation of human macrophages is its dependence on the presence of serum (and, in particular, LBP) in the culture medium. We therefore investigated whether these effects also occurred with NK cells in serum-free conditions in the culture medium. For this purpose, we performed experiments with R- and S-LPS using the AIM-V serum-free medium. The absence of serum in culture medium did not abrogate the stimulating effect of S-LPS (Figure 2(a)). Thus, these findings showed that the presence of the serum has not been required for the interaction of S-LPS with NK cell.

### 3.3. S-LPS from Different Strains of E. coli Activates NK Cells with a Comparable Efficiency

Next, we investigated the role of the presence or absence of the O-antigen in the LPS-induced activation of NK cells. We have tested S-LPS from three different *E. coli* strains (O55, O111, and O127, see also Figures 1(b) and 2(c)) for their ability to induce IFN*γ* production by NK cells. The description of structural features of their oligosaccharide repeating units can be found here:

(i): O55: http://nevyn.organ.su.se/ECODAB/showDetails.php?idSerogroup=72
(ii): O111: http://nevyn.organ.su.se/ECODAB/showDetails.php?idSerogroup=64
(iii): O127: http://nevyn.organ.su.se/ECODAB/showDetails.php?idSerogroup=112

Besides the O-antigen, the chosen LPS preparations differed in structure of their outer core: O55 and O111 refer to R3 type and O127 refers to R2. All three S-LPS preparations effectively induced IFN*γ* production in NK cells (Figure 2(b)). Lipopolysaccharides O55 and O111 demonstrated similar effects in different experiments; influence of O127 has been significantly lower. The differences in observed effects could be linked with the outer core and/or O-antigen structures. Thus, the structure of saccharide part of LPS influenced the magnitude of increase in IFN*γ* production by NK cells.

### 3.4. S-LPS Lacking Fatty Acid Residues Fails to Stimulate NK Cells

There was also a possibility that the stimulation of IFN*γ* production by LPS was linked exclusively with the saccharide but not the lipid moiety of the LPS molecule. To test this, we investigated whether LPS lacking fatty acid chains retained the ability to increase IFN*γ* production by NK cells. Experiments were performed using “detoxified” (deacylated) LPS from the same strains as used above, i.e., O55 and O111. According to the information provided by the manufacturer, the removal of the fatty acid portions of lipid A (deacylated LPS) decreases the endotoxin level by roughly 10,000 times in detoxified LPS compared to the parent LPS. The electrophoretic analysis showed no bands in the samples with detoxified LPS (Figure 1(b)). The absence of bands of detoxified LPS on PAGE gel indirectly confirmed that these LPS preparations were effectively deacylated. In all experiments performed, the detoxified LPS had no significant effect on IFN*γ* production by NK cells (Figure 2(b)), while their parent LPS (O55 and O111) demonstrated high effects. This finding showed that the presence of fatty acid chains is necessary for the stimulating effect of LPS on NK cells. Taken together, our data showed that both lipid and saccharide parts of LPS molecule are important for the IFN*γ* production by NK cells.

### 3.5. The Activating Effect of LPS on NK Cells Is Not Caused by Admixtures of DCs in NK Cell Fractions

**Figure 3:**
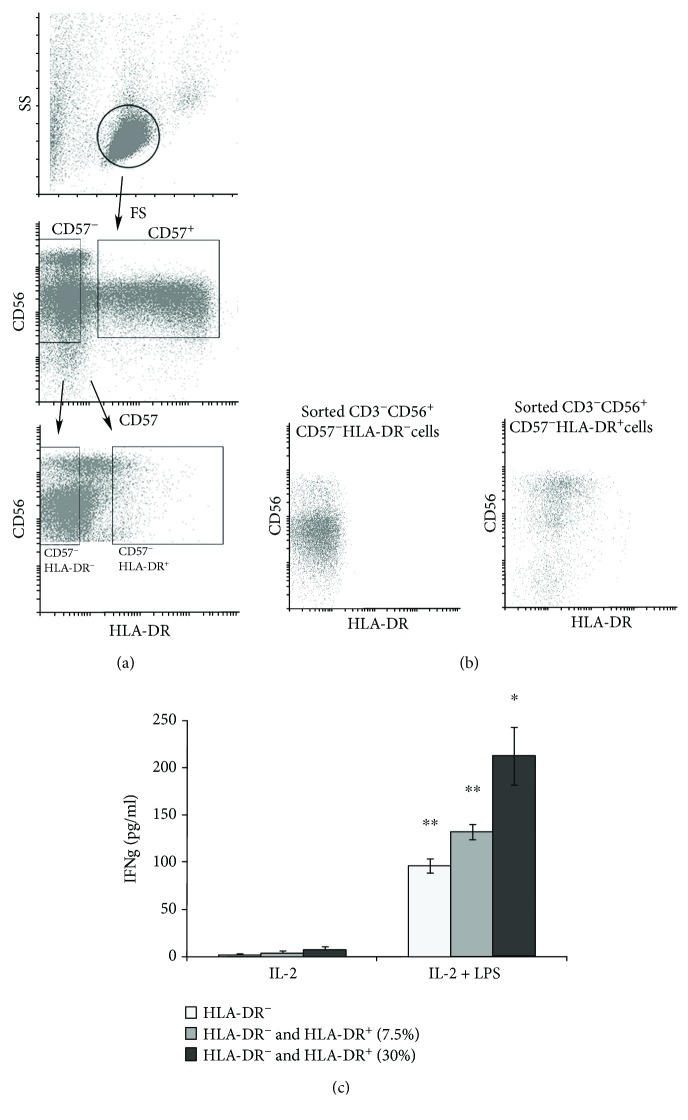
The effects of LPS have not been caused by a presence of DCs in NK cell fractions. (a) Gating strategy for the sorting of NK cell subpopulations. (b) Sorted CD57^+^ and CD57^–^ NK cells. (c) Sorted CD57^–^HLA-DR^–^ NK cells were able to respond independently to the LPS stimulation of HLA-DR^+^ cell presence. CD57^–^HLA-DR^–^ and CD57^–^HLA-DR^+^ NK cells were isolated by cell sorting, then mixed in indicated proportions and incubated with IL-2 (500 U/ml) and S-LPS (5 *μ*g/ml) overnight. Results of one of three experiments performed on the blood samples from different donors are shown. Data are mean ± SD of triplicate wells.

These studies were performed with a magnetic enrichment method for separation of the NK cells. Previous studies have shown that using this method can lead to obtaining of NK cell preparations containing significant numbers of dendritic cells (DCs), a population referred to as slanDCs. According to Costantini et al. [15], the presence of slanDCs caused the observed stimulating effect of LPS on NK cell fraction. SlanDCs express HLA-DR, as do a portion of NK cells in these enriched preparations. To verify whether the presence of the HLA-DR-expressing non-NK cells caused the observed activating LPS action, we further purified the magnetically enriched NK populations into HLA-DR negative and positive subpopulations using fluorescence-activated cell sorting. Firstly, NK cells were purified using the Miltenyi Biotec kit. Secondly, they were stained by antibodies to CD3, CD56, CD57, and HLA-DR. From the CD3^–^CD56^+^CD57^–^ NK cells HLA-DR negative and positive cells have been sorted (Figure 3(a)). Both collected NK cell fractions were CD57 negative (Figure 3(b)), previously shown to respond to the LPS [16]. Thirdly, the fractions of HLA-DR negative and positive CD57^–^ NK cells were mixed in different proportions (HLA-DR^–^ cells only, HLA-DR^–^ + HLA-DR^+^ (7.5%) and HLA-DR^–^ + HLA-DR^+^ (30%)) in RPMI+10% FCS culture medium, then IL-2 with S-LPS was added. Next, after overnight incubation, the IFN*γ* level was evaluated.

As shown in Figure 3(c), all three mixtures of cells demonstrated the significant LPS-induced increase in IFN*γ* production. LPS could therefore stimulate NK cells independently of the amount of HLA-DR^+^ cells in the fraction, including slanDCs. This further supports a model of direct (independent from other immune cells) action of LPS on NK cells. The amount of the HLA-DR^+^ NK cells in the NK cell mixtures increased the basal and LPS-induced IFN*γ* production by NK cells (Figure 3(c)). This observation can be linked with the features of HLA-DR^+^ NK cells. Expression of HLA-DR on NK cells has been shown to correlate with an activated status of these cells, evidenced by an increase in cytokine production and cytotoxicity [17–19].

## 4. Discussion

To date, the mechanism of LPS-induced cell activation is well described for macrophages. It includes the interaction of exogenous LPS with TLR4 receptor complex with the help of LBP and coreceptor CD14 [9]. LPS has been found to be able to activate human NK cells, although they do not express or express very low level of TLR4 on the cell surface [3–5], and the surface expression of CD14 has not been detected too (data not shown). This work has revealed that the conditions for the LPS-induced NK cell activation also have some differences from the classical model.

First of all, only S- but not R-forms of LPS were able to stimulate IFN*γ* production in human NK cells. With regard to the R-forms, it is important to note that due to the lack of long polysaccharide fragment (O-antigen), the same concentration of the R-LPS preparations contained several times more molecules able to trigger TLR4 (i.e., containing lipid A fragment) than the S-LPS preparations. Therefore, the molar concentration of the TLR4 agonists is several times higher in R-LPS preparations. Herewith, the stimulating effect of R-LPS on IFN*γ* production was negligible. Thus, the LPS preparation with higher concentration of the TLR4 agonists was notably less effective for NK cells than that with lower concentration, as opposed to the situation with macrophages [10].

Next, LPS was able to increase IFN*γ* production irrespectively to the presence or absence of serum in the culture media. This observation can evidence that LBP plays no significant role in the triggering of the NK cell activity by LPS. Also, LPS lacking fatty acid chains did not influence IFN*γ* production, indicating that the NK cell sensor of LPS should interact with the lipid A part of LPS molecule, like in the classical model.

In summary, our results indicate that the presence of both saccharide and lipid parts of LPS molecule is required for the observed effect of LPS on NK cells. Any of the parts alone did not activate NK cells, but the lack of any of them in LPS nearly fully abrogated the stimulating action of the lipopolysaccharide. It can be suggested that the lipid component of the exogenous LPS is involved in transient capturing the molecule by the NK cell plasma membrane that enables the saccharide-dependent effect of LPS on the NK cell.

## 5. Conclusions

Taken together, our data depicts the number of specific characteristics of the LPS action on human NK cells. The fact that LPS can stimulate natural killer cells is important not only for investigation of the role of NK cells in sepsis but also in *in vitro* studies of the effects of recombinant proteins, expressed in *E.coli*, on human NK cells.

## Data Availability

No data were used to support this study.

## Supplementary Materials

Supplementary Materials Pages 1-7 contain the certificates of analysis of lipopolysaccharide preparations purchased by Sigma-Aldrich which have been used in the current work. The preparations were the following: 1: wild-type E. coli LPS, strain O55:B5; 2: wild-type E. coli LPS, strain O111:B4; 3: wild-type E. coli LPS, strain O127:B8; 4: wild-type E. coli LPS, strain O55:B5, partially delipidized by alkaline hydrolysis; 5: wild-type E. coli LPS, strain O111:B4, partially delipidized by alkaline hydrolysis; 6: Rd mutant E. coli LPS; and 7: Ra mutant E. coli LPS. Pages 8 and 9 contain the results of mass spectrometry analysis of certain preparations of LPS: MS analysis of both wild-type and delipidized LPSs, strain O55 and 9: MS analysis of both wild-type and delipidized LPSs, strain O111.
