# Supplementary material for: The Role of O-Antigen in LPS-Induced Activation of Human NK Cells
Source: J Immunol Res. 2019 May 20;2019:3062754. doi: 10.1155/2019/3062754 (PMC6545784; doi:10.1155/2019/3062754)
Supplement: Supplementary Materials — Pages 1-7 contain the certificates of analysis of lipopolysaccharide preparations purchased by Sigma-Aldrich which have been used in the current work. The preparations were the following: 1: wild-type E. coli LPS, strain O55:B5; 2: wild-type E. coli LPS, strain O111:B4; 3: wild-type E. coli LPS, strain O127:B8; 4: wild-type E. coli LPS, strain O55:B5, partially delipidized by alkaline hydrolysis; 5: wild-type E. coli LPS, strain O111:B4, partially delipidized by alkaline hydrolysis; 6: Rd mutant E. coli LPS; and 7: Ra mutant E. coli LPS. Pages 8 and 9 contain the results of mass spectrometry analysis of certain preparations of LPS: MS analysis of both wild-type and delipidized LPSs, strain O55 and 9: MS analysis of both wild-type and delipidized LPSs, strain O111. [file 3062754.f1.pdf]

SIGMA-ALDRICH®

sigma-aldrich.com

3050 Spruce Street, Saint Louis, MO 63103, USA
Website: www.sigmaaldrich.com
Email USA: techserv@sial.com
Outside USA: eurtechserv@sial.com

Certificate of Analysis

Product Name:
Lipopolysaccharides from Escherichia coli O55:B5 - purified by ion-exchange chromatography, TLR ligand tested

**Product Number:**	**L4524**
**Batch Number:**	**045M4024V**
Brand:	SIGMA
Storage Temperature:	Store at 2 - 8 °C
Quality Release Date:	16 FEB 2015
Recommended Retest Date:	FEB 2021

Test	Specification	Result
Appearance (Form)	Lyophilized Powder	Lyophilized Powder
Appearance (Colour)	White to White w/ Yellow Cast	White
Solubility (Solvent)	Water	Water
Solubility (Conc)	4.90 - 5.10 mg/ml	5.00 mg/ml
Solubility (Turbidity)	Hazy	Hazy
Solubility (Color)	Colorless to Light Yellow	Colorless
Protein Content (Method)	Lowry-TCA	Lowry-TCA
Prot. Content (% Protein)	≤ 1.00 %	0.93 %
Potency (Sample EU/mg)	≥ 500000 EU/mg	1200000 EU/mg
UV (% RNA)	≤ 1.00 %	0.00 %

Theo Ackermann

Theo Ackermann PhD MScEng CQM
Manager, Quality and Regulatory Affairs
Jerusalem, Israel IL

Sigma-Aldrich warrants, that at the time of the quality release or subsequent retest date this product conformed to the information contained in this publication. The current Specification sheet may be available at Sigma-Aldrich.com. For further inquiries, please contact Technical Service. Purchaser must determine the suitability of the product for its particular use. See reverse side of invoice or packing slip for additional terms and conditions of sale.

SIGMA-ALDRICH
sigma-aldrich.com
3050 Spruce Street, Saint Louis, MO 63103, USA
Website: www.sigmaaldrich.com
Email USA: techserv@sial.com
Outside USA: eurtechserv@sial.com

Certificate of Analysis

Product Name:
Lipopolysaccharides from Escherichia coli 0111:B4 - purified by ion-exchange chromatography, TLR ligand tested

**Product Number:**	**L3024**
**Batch Number:**	**025M4041V**
Brand:	SIGMA
Storage Temperature:	Store at 2 - 8 °C
Quality Release Date:	14 DEC 2014
Recommended Retest Date:	DEC 2020

Test	Specification	Result
Appearance (Form)	Lyophilized Powder	Lyophilized Powder
Appearance (Colour)	White to White w/ Yellow Cast	White
Solubility (Solvent)	Water	Water
Solubility (Conc)	4.90 - 5.10 mg/ml	5.00 mg/ml
Solubility (Turbidity)	Hazy	Hazy
Solubility (Color)	Colorless to Light Yellow	Colorless
Protein Content (Method)	Lowry-TCA	Lowry-TCA
Prot. Content (% Protein)	≤ 1.00 %	0.64 %
UV (% RNA)	≤ 1.00 %	0.00 %
Potency (Sample EU/mg)	≥ 500000 EU/mg	1200000 EU/mg
Storage Conditions	Desiccated	Desiccated

Theo Ackermann

Theo Ackermann PhD MScEng CQM
Manager, Quality and Regulatory Affairs
Jerusalem, Israel IL

Sigma-Aldrich warrants, that at the time of the quality release or subsequent retest date this product conformed to the information contained in this publication. The current Specification sheet may be available at Sigma-Aldrich.com. For further inquiries, please contact Technical Service. Purchaser must determine the suitability of the product for its particular use. See reverse side of invoice or packing slip for additional terms and conditions of sale.

SIGMA-ALDRICH®

sigma-aldrich.com

3050 Spruce Street, Saint Louis, MO 63103, USA
Website: www.sigmaaldrich.com
Email USA: techserv@sial.com
Outside USA: eurtechserv@sial.com

Certificate of Analysis

Product Name:
Lipopolysaccharides from Escherichia coli 0127:B8 - purified by ion-exchange chromatography, TLR ligand tested

**Product Number:**	**L5024**
**Batch Number:**	**046M4083V**
Brand:	SIGMA
Storage Temperature:	Store at 2 - 8 ℃
Quality Release Date:	27 MAR 2016
Recommended Retest Date:	MAR 2022

**Test**	**Specification**	**Result**
Appearance (Form)	Lyophilized Powder	Lyophilized Powder
Appearance (Colour)	White to White w/ Yellow Cast	White
Solubility (Solvent)	Water	Water
Solubility (Conc)	4.90 - 5.10 mg/ml	5.00 mg/ml
Solubility (Turbidity)	Faint Hazy to Hazy	Hazy
Solubility (Color)	Colorless to Faint Yellow	Colorless
Protein Content (Method)	Lowry-TCA	Lowry-TCA
Prot. Content (% Protein)	$\leq$ 1.00 %	0.35 %
UV (% RNA)	$\leq$ 1.00 %	0.10 %
Potency (Sample EU/mg)	$\geq$ 500000 EU/mg	> 1200000 EU/mg
Water (by Karl Fischer)	$\geq$ 0.00 %	11.21 %

Theo Ackermann PhD MScEng CQM
Manager, Quality and Regulatory Affairs
Jerusalem, Israel IL

Sigma-Aldrich warrants, that at the time of the quality release or subsequent retest date this product conformed to the information contained in this publication. The current Specification sheet may be available at Sigma-Aldrich.com. For further inquiries, please contact Technical Service. Purchaser must determine the suitability of the product for its particular use. See reverse side of invoice or packing slip for additional terms and conditions of sale.

SIGMA-ALDRICH®

sigma-aldrich.com

3050 Spruce Street, Saint Louis, MO 63103, USA
Website: www.sigmaaldrich.com
Email USA: techserv@sial.com
Outside USA: eurtechserv@sial.com

Certificate of Analysis

Product Name:
Lipopolysaccharides from Escherichia coli O55:B5 – Detoxified

Product Number: **L9023**
Batch Number: **016M4009V**
Brand: SIGMA
Storage Temperature: Store at 2 - 8 °C
Quality Release Date: 26 NOV 2015
Recommended Retest Date: NOV 2021

Test	Specification	Result
Appearance (Form)	Lyophilized Powder	Lyophilized Powder
Appearance (Colour)	White to Yellow/Tan	White
Solubility (Solvent)	Water	Water
Solubility (Conc)	4.90 - 5.10 mg/ml	5.00 mg/ml
Solubility (Turbidity)	Faint Hazy to Hazy	Faint Hazy
Solubility (Color)	Colorless to Yellow or Tan	Colorless
Protein Content (Method)	Lowry-TCA	Lowry-TCA
Prot. Content (% Protein)	≤ 3.00 %	0.68 %
Potency (Sample EU/mg)	≤ 1000 EU/mg	900 EU/mg

Theo Ackermann

Theo Ackermann PhD MScEng CQM
Manager, Quality and Regulatory Affairs
Jerusalem, Israel IL

Sigma-Aldrich warrants, that at the time of the quality release or subsequent retest date this product conformed to the information contained in this publication. The current Specification sheet may be available at Sigma-Aldrich.com. For further inquiries, please contact Technical Service. Purchaser must determine the suitability of the product for its particular use. See reverse side of invoice or packing slip for additional terms and conditions of sale.

SIGMA-ALDRICH
sigma-aldrich.com

3050 Spruce Street, Saint Louis, MO 63103, USA
Website: www.sigmaaldrich.com
Email USA: techserv@sial.com
Outside USA: eurtechserv@sial.com

Certificate of Analysis

Product Name:
Lipopolysaccharides from Escherichia coli 0111:B4 - Detoxified

Product Number: **L3023**
Batch Number: **055M4086V**
Brand: SIGMA
Storage Temperature: Store at 2 - 8 ℃
Quality Release Date: 26 MAY 2015
Recommended Retest Date: MAY 2021

Test	Specification	Result
Appearance (Form)	Lyophilized Powder	Lyophilized Powder
Appearance (Colour)	White to Yellow and Faint Beige to Beige	Light Beige
Solubility (Solvent)	Water	Water
Solubility (Conc)	4.90 - 5.10 mg/ml	5.00 mg/ml
Solubility (Turbidity)	Very Slightly Hazy to Hazy	Very Slightly Hazy
Solubility (Color)	Faint Yellow to Tan	Light Tan
Potency (Sample EU/mg)	≤ 1000 EU/mg	< 1000 EU/mg
Handling Precautions	Hygroscopic	Hygroscopic

Theo Ackermann PhD MScEng CQM
Manager, Quality and Regulatory Affairs
Jerusalem, Israel IL

Sigma-Aldrich warrants, that at the time of the quality release or subsequent retest date this product conformed to the information contained in this publication. The current Specification sheet may be available at Sigma-Aldrich.com. For further inquiries, please contact Technical Service. Purchaser must determine the suitability of the product for its particular use. See reverse side of invoice or packing slip for additional terms and conditions of sale.

3050 Spruce Street, Saint Louis, MO 63103, USA
Website: www.sigmaaldrich.com
Email USA: techserv@sial.com
Outside USA: eurtechserv@sial.com

Certificate of Analysis

Product Name:
Lipopolysaccharides (rough strains) from Escherichia coli F583 (Rd mutant)

**Product Number:**	**L6893**
**Batch Number:**	**042M4093V**
Brand:	SIGMA
Storage Temperature:	Store at 2 - 8 °C
Quality Release Date:	26 MAR 2012
Recommended Retest Date:	MAR 2015

Test	Specification	Result
Appearance (Form)	Lyophilized Powder	Lyophilized Powder
Appearance (Colour)	White to White w/ Yellow Cast	White
Solubility (Solvent)	Water	Water
Solubility (Conc)	4.90 - 5.10 mg/ml	5.00 mg/ml
Solubility (Turbidity)	Slightly Hazy to Turbid	Hazy
Solubility (Color)	Colorless to Faint Yellow	Colorless
Protein Content (Method)	Lowry-TCA	Lowry-TCA
Prot. Content (% Protein)	≤ 3.00 %	2.80 %
Potency (Sample EU/mg)	≥ 500000 EU/mg	600000 EU/mg

Theo Ackermann PhD MScEng CQM
Manager, Quality and Regulatory Affairs
Jerusalem, Israel IL

Sigma-Aldrich warrants, that at the time of the quality release or subsequent retest date this product conformed to the information contained in this publication. The current Specification sheet may be available at Sigma-Aldrich.com. For further inquiries, please contact Technical Service. Purchaser must determine the suitability of the product for its particular use. See reverse side of invoice or packing slip for additional terms and conditions of sale.

SIGMA-ALDRICH
sigma-aldrich.com
3050 Spruce Street, Saint Louis, MO 63103, USA
Website: www.sigmaaldrich.com
Email USA: techserv@sial.com
Outside USA: eurtechserv@sial.com

Certificate of Analysis

Product Name:
Lipopolysaccharides (rough strains) from Escherichia coli EH100 (Ra mutant)

Product Number: **L9641**
Lot Number: **071M4120V**
Brand: SIGMA
Storage Temperature: Store at 2-8 DEGREE C
Quality Release Date: 21 JUN 2011
Recommended Retest Date: JUN 2014

Test	Specification	Result
Appearance (Form)	Lyophilized Powder	Lyophilized Powder
Appearance (Colour)	White to Light Yellow/Tan	Off-White
Solubility (Solvent)	Water	Water
Solubility (Conc)	1.90 - 2.10 mg/ml	2.00 mg/ml
Solubility (Turbidity)	Faint Hazy to Hazy	Slightly Hazy
Solubility (Color)	Colorless to Faint Yellow	Colorless
Protein Content (Method)	Lowry-TCA	Lowry-TCA
Prot. Content (% Protein)	≤ 3.00 %	2.42 %
Potency (Sample EU/mg)	≥ 500000 EU/mg	1200000 EU/mg

Theo Ackermann

Theo Ackermann PhD MScEng CQM
Manager, Quality and Regulatory Affairs
Jerusalem, Israel IL

Sigma-Aldrich warrants, that at the time of the quality release or subsequent retest date this product conformed to the information contained in this publication. The current Specification sheet may be available at Sigma-Aldrich.com. For further inquiries, please contact Technical Service. Purchaser must determine the suitability of the product for its particular use. See reverse side of invoice or packing slip for additional terms and conditions of sale.

Comment 1

Comment 2

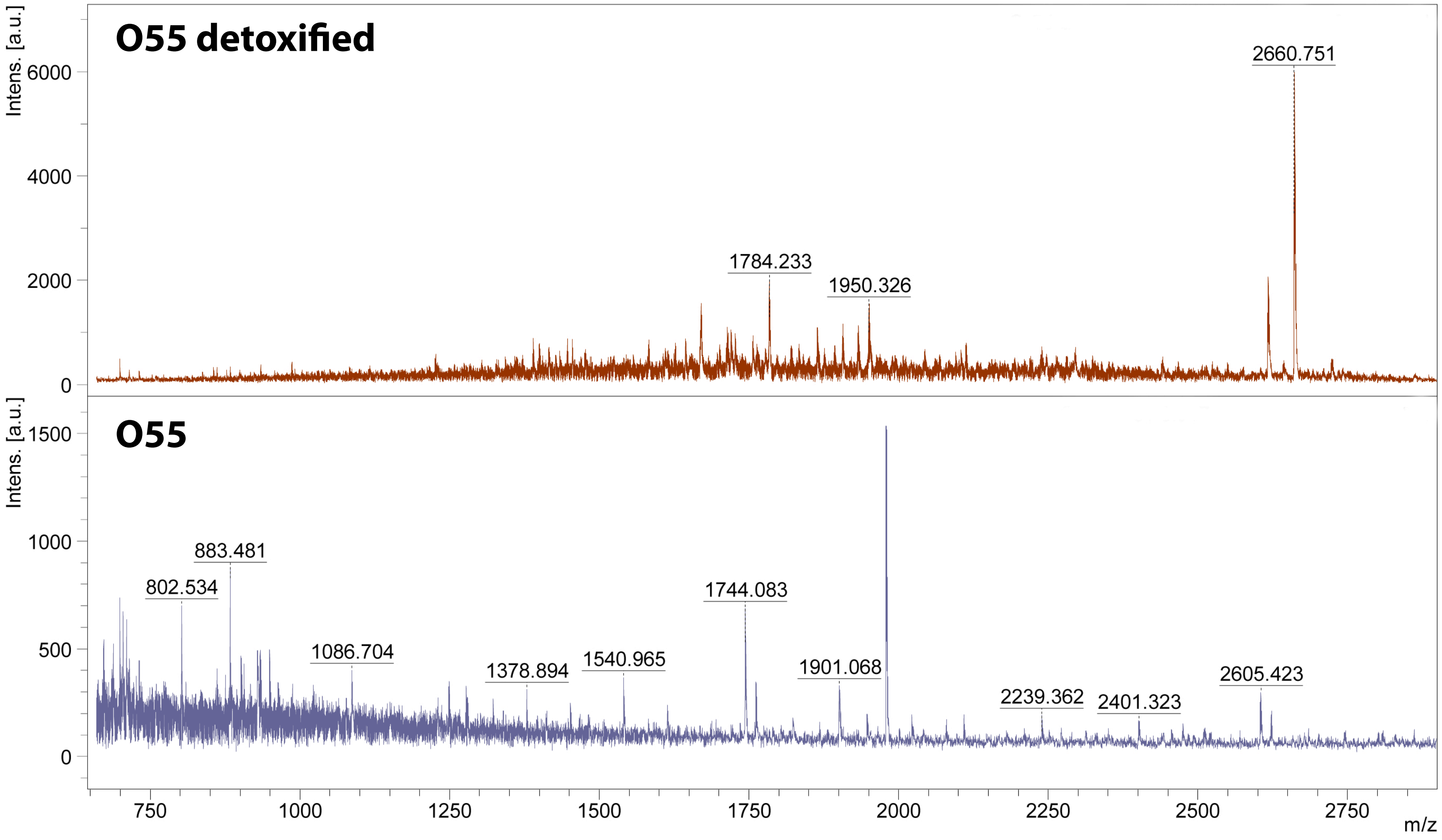

m/z	S/N	Area
802.534	3.1	208
883.481	3.8	297
1086.704	2.1	139
1248.791	1.9	118
1378.894	2.2	109
1540.965	3.0	133
1744.083	6.0	742
1901.068	3.5	180
1979.252	18.0	1876
2239.362	1.4	73
2401.323	2.3	75
2605.423	4.2	145

Comment 1

Comment 2

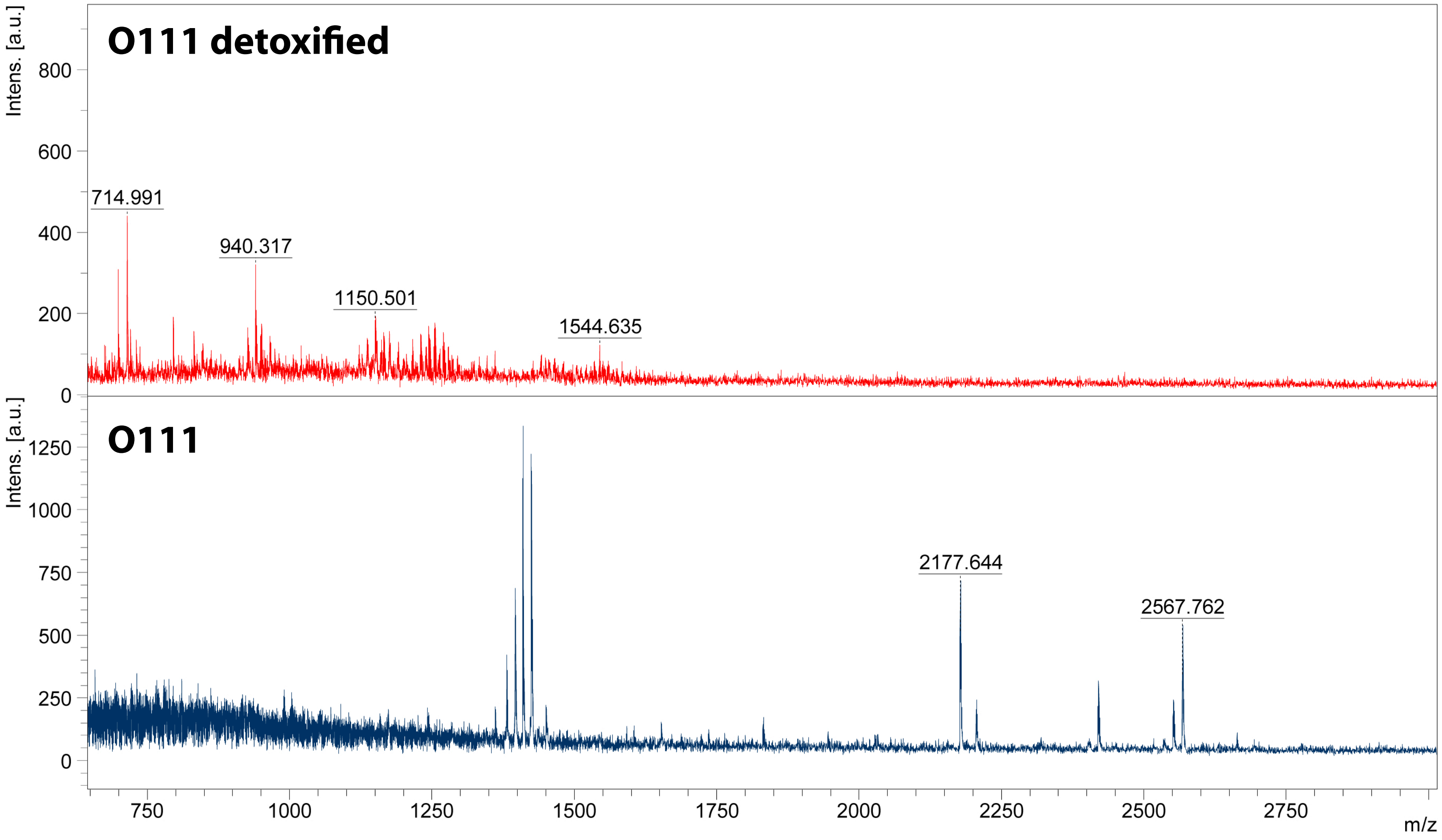

m/z	S/N	Area
699.002	5.5	61
714.991	7.9	81
940.317	5.6	73
1150.501	3.4	43
1216.529	2.6	37
1254.535	3.2	49
1270.518	3.0	36
1544.635	2.8	21
